# P-cadherin expression and survival rate in oral squamous cell carcinoma:an immunohistochemical study

**DOI:** 10.1186/1471-2407-5-63

**Published:** 2005-06-21

**Authors:** Lorenzo Lo Muzio, Giuseppina Campisi, Antonio Farina, Corrado Rubini, Giuseppe Pannone, Rosario Serpico, Gregorio Laino, Alfredo De Lillo, Francesco Carinci

**Affiliations:** 1 Department of Surgical Sciences, University of Foggia, Foggia, Italy; 2 Department of Dental Sciences "G. Messina", University of Palermo, Palermo, Italy; 3 Institute of Histology, University of Bologna, Bologna, Italy; 4 Institute of Pathology, University of Ancona, Ancona, Italy; 5 Department of Dental Sciences, Second University of Naples, Naples, Italy; 6 Institute of Maxillofacial Surgery, University of Ferrara, Ferrara, Italy

## Abstract

**Background:**

P-cadherin (P-cad) is a transmembrane molecule involved in the cell-cell adhesion and similar to E-cadherin (E-cad), but less investigated in oncology, especially in *in vivo *studies. Aims of the present study were to assess the prevalence of P-cad expression in oral squamous cell carcinoma (OSCC) and to verify whether P-cad can be considered a marker of prognosis in patients with OSCC.

**Methods:**

In a retrospective study, a cohort of 67 OSCC patients was investigated for P-cad expression and its cellular localization by immunohistochemistry; some respective healthy margins of resection were similarly investigated as standard controls. After grouping for P-cad expression, OSCCs were statistically analyzed for the variables age, gender, histological grading (G), TNM, Staging, and overall survival rate. Univariate and multivariate analyses were performed.

**Results:**

37 cases (55.2%) of OSCC showed membranous/cytoplasmic positivity for P-cad, whereas 30 (44.8 %) were negative. Although with some differences in membranous vs cytoplasmic localization of P-cad in OSCC with different G, no statistical association was found between P-cad expression and any variables considered at baseline. In terms of prognostic significance, P-cad non expression was found to have an independent association with poorer overall survival rate than P-cad expressing group (P = 0.056); moreover, among P-cad +ve patients the best prognosis was for those OSCC with membranous (P < 0.0001) than those with cytoplasmic P-cad expression.

**Conclusion:**

On the basis of these results, it is possible to suggest P-cad as an early marker of poor prognosis. The abnormal or lack of P-cad expression could constitute an hallmark of aggressive biological behavior in OSCC

## Background

Invasive OSCC, in spite of improved therapeutic procedures, actually show a generally poor prognosis since its local aggressiveness and metastases. In particular, the incidence of lymph node metastases has been found to be significantly associated with several factors; among these, not only macroscopic parameters, such as clinical stage, localization and thickness of primary tumours [1-10], but also microscopic-molecular parameters from differentiation of tumoral cells up to their skill for adherence [11-14]. Recently, many investigations have been performed in this latter direction, until to know that intercellular adhesiveness is mediated by a family of glycoproteins named cadherins [15]. This family is composed of an extra-cellular domain, involved in Ca^++^-dependent homophilic binding to adjacent cells, a trans-membrane domain, and an intra-cellular domain which binds to proteins called catenins [16]. In epithelial cells, this adhesiveness is mediated by epithelial-cadherin (E-cad), a 120-kd transmenbrane glycoprotein, localized mainly in the zonula adherens junctions. The cadherin family includes also other members: neural-cadherin (N-cadherin) [17], placental cadherin (P-cad) [18] and liver cell adhesion molecule (L-CAM), and more than 20 cadherins have been described in the central nervous system, liver and vascular endothelial cells and in other tissues and organs [19,20]. In particular, P-cad is a protein homologous to E-cad. E-cadherin is involved in the adherens type of intercellular junctions of keratinocytes, while P-cad is detected on the cell-cell contact surface of basal keratinocytes in normal mouse and human epidermis and cells migrating into the suprabasal compartiment down-regulate P-cad expression. Both these molecules interact with cytoskeleton by alpha-catenin.

P-cad is expressed in mouse placenta [18], epithelia [21], the basal cell of the skin [22,23], playing an important role in the morphogenesis of epidermis and skin appendage [22,24,25]. The expression of P-cad in epithelial tissues appears to identify cell populations with proliferative activity, and its expression decreases as cells differentiate [23,26].

The possible role exerted by cadherins in human carcinogenesis has been suggested by a number of studies [27,28]. Down-regulation of E-cad was reported to be directly related to invasiveness and progression of many human epithelial tumours [28], including oral squamous cell carcinomas (OSCC) [29].

While E-cad expression has been extensively studied in many forms of human cancers, including OSCC [27,30-40], less is known about the expression levels of P-cad in human cancers [23,41-51] and, particularly, in OSCCs *in vivo *[35,39,40,52].

Aims of the present study were to assess the prevalence of P-cad expression in oral squamous cell carcinoma (OSCC) and to verify whether P-cad can be considered a marker of prognosis in patients with OSCC.

## Methods

Sixty-seven patients affected by histologically proven OSCC were consecutively recruited among those surgically treated in a multicentric study between January 1992 and December 1997. The patients, never treated before for OSCC, included 45 males (67.2%) and 22 females (32.8%). They ranged in age from 18 to 87 years (median age 65 years) at the time of admission; 20 (29.8%) had neck nodes, and none had evidence of distant metastases. Tumours were classified according to U.I.C.C. 2000 classification *(UICC 2000)*, reaching to the following Stage Grouping: Stage I for 30 OSCC, II for 15, III for 11 and IV for 11.

Although recruited in different centres, all of OSCCs were treated according to the common and current Guidelines dedicated[53]. In particular, sAlthough treated in deifferent centers, all odf OSCCs were Alkjkkhhhpurgery on T was the treatment of choice and always performed at the initial course of the protocol with curative intent (i.e. only tumour resections in safe margins were done). When radiotherapy was considered useful, it was usually done 3 weeks after surgery, with external beam and the dose was equivalent to 60 or 65 Gy in 6 or 7 weeks. Chemotherapy, when prescribed, consisted of cisplatin (100 mg/m^2 ^body-surface area) given as intravenous infusion followed by continuous 24-hour intravenous infusion of fluorouracil (1,000 mg/m^2 ^per day) for five days. globally up to 3 cycles. All patients were followed up and examined on a monthly basis for the first year after treatment, every 2 months for the second year, and every 3 months thereafter. At our baseline, an overall disease-specific survival was calculated at 72 months for all patients plus cases censored (for death).

### Immunohistochemistry

5 μm serial sections from routinely formalin-fixed paraffin-embedded blocks were cut for each case, and one section stained with haematoxylin-eosin (H.E.) was used to confirm the histopathological diagnosis. Only sections showing sufficient epithelium to assess 1000 cells were considered for this study. In addition, some microscopically healthy specimens of oral mucosa from juxtaposed sites to OSCC went to similar investigation as standard controls.

Immunocytochemistry was then performed on the remaining sections mounted on poly-L-lysine-coated glass slides. Endogenous peroxidase was quenched by incubating the sections for 20 minutes with 0,3% hydrogen peroxide in methanol. To improve the staining pattern, the sections were boiled three times for 3 minutes in 10 mM citrate buffer as an antigen retrieval method. In order to prevent non-specific binding of antibodies, sections were then pre-incubated with non-immune mouse serum (1:20; Dakopatts, Hamburg, Germany) and diluted in PBS/BSA (1%) for 25 minutes at room temperature. After washing twice with Tris-HCl buffer, primary antibodies were applied. As positive controls, the immunoreactivity of 5 normal skin sections from leg was evaluated. A negative control was also performed in each run by substituting primary antibodies with non-immune serum (DAKO Antibody Diluent, Dakopatts, Hamburg, Germany). All the slides were washed twice in Tris-HCl buffer between each step. Commercially available mouse monoclonal IgG antibody against P-cad (Transduction Laboratories, Lexington, Kentucky, U.S.A.), packaged at 0.25 mg/ml, was used at a dilution of 1:300. Then two methods were applied: Labeled streptavidin-biotin-peroxidase technique (LSAB-HRP) and Labeled Streptavidin-biotin-alkaline phosphatase technique (LSAB-AP). In LSAB-HRP technique sequential 20-minute incubation with biotinylated linking antibody and horseradish peroxidase-labeled streptavidin (Dako LSAB + kit, HRP) were performed at room temperature. The peroxidase activity was developed by incubation with 3.3'-diaminobenzidine (DAB, Vector Laboratories, Burlinghame, USA) as a substrate chromogen solution. The slides were then counterstained with hematoxylin. In LSAB-AP technique sequential 20-minute incubations with biotinylated anti-mouse immunoglogulins and streptavidin conjugated to alkaline phosphatase were performed. Finally, a new fast red substrate system (K0597, Dako, Glostrup, Denmark) was applied as a chromogen solution. The specificity of this antibody has been described in the literature [42].

### Evaluation of immunostaining

The number of P-cad-expressing tumour cells was estimated as a percentage of the final number of 500 neoplastic cells of each case, and scored in two categories: score 0 (≤ 5% of cells were positive), score 1 (P-cad expression in > 5 of cells %). The expansion of P-cad-positive cells in the spinous layer was defined as anomalous P-cad expression [47].

### Statistical analysis

#### Univariate analysis

Differences between P-cad expression values and the variables considered were analysed by means of Student-Newmann-Keuls' test (simple or in multiple comparison) and by ANOVA. The difference was considered significant when p-value was ≥ 0.05. Disease-specific survival curves were calculated according to the product-limit method (Kaplan-Meier algorithm). Time zero was defined as the date of the patient's initial diagnosis. Patients who are still alive were included in the total number at risk of death only up to the time of their last follow-up. Therefore, the survival rate only changed when death occurred. Patients dead during the follow-up period (i.e. 72 months) were considered as censored. The calculated survival rate was the maximum estimate of the true survival curve. Log rank test was used to compare survival curves, generated by stratifications for a variable of interest.

#### Cox regression analysis

Afterwards, Cox regression analysis was applied to determine the single contribution of covariates on survival rate. Cox regression analysis compares survival data while taking into account the statistical value of independent variables, such as age and sex, on whether or not an event (i.e. death) is likely occur. If the associated probability was less then 5% (p < .05), the difference was considered statistically significant. In the process of doing the regression analysis, odds ratio (OR) and 95% confidence interval (CIs) were calculated. Stepwise Cox analysis allowed us to detect the variables most associated to survival.

## Results

### P-cad expression

**Figure 1:**
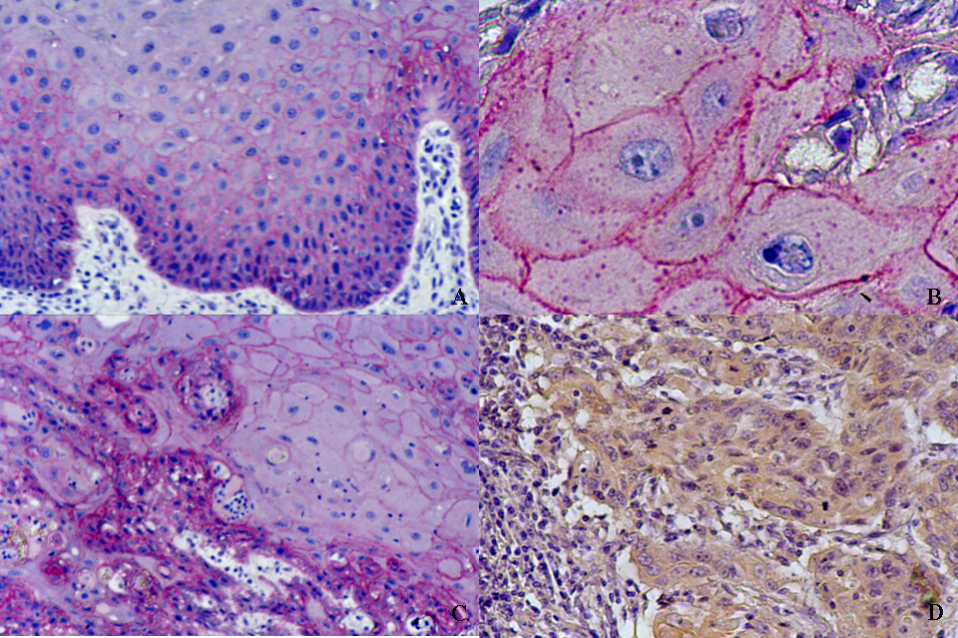
a) Strong basal-parabasal membranous expression of P-cadherin in oral hyperplastic epithelium (LSAB-AP, nuclear counterstaining with haematoxylin, ×106); b) Membranous P-cadherin expression in oral moderately-differentiated SCC (LASB-AP, ×400); c) Membranous expression of P-cadherin in an area of stromal infiltration from moderately-differentiated OSCC (LASB-AP, nuclear counterstaining with haematoxylin, ×160); d) Cytoplasmic expression of P-cadherin in a case of lowly differentiated OSCC infiltrating stroma (LSAB-HRP, ×250)

First of all, the paradigmatic P-cad expression was obtained from the standard controls (Fig. 1a): in these, in fact, only a membranous staining was observed at the basal layer of histologically normal oral epithelium; predominantly on the membrane of only a thin line of cells basally located, with occasionally moderate parabasal staining. The intensity of staining for P-cadherin progressively reduced from basal to parabasal layers and stopped in the spinous layer. No staining for P-cadherin was observed in the upper layer.

37 cases (55.2%) OSCC showed membranous/cytoplasmic positivity for P-cad (Group P-cad +ve)(Table 1), whereas 30 (44.8 %) were found negative (Group P-cad -ve). When examined the cell staining pattern of positive cases, 25 cases showed a prevalent membranous pattern (Fig. 1b-c), while 12 had a prevalent cytoplasmic pattern (Fig. 1d). Worthy of note, within the Group P-cad +ve, dedifferentiated areas showed both membranous and cytoplasmic P-cad up-regulation: well-differentiated (G1) oral carcinomas showed P-cad up-regulation, while P-cad expression homogeneously reduced in scarcely differentiated oral squamous cell carcinomas (G3), and it shifted to membranous/cytoplasmic co-localization, predominantly cytoplasmic in distribution, or alternatively was absent in a large numbers of cells. Although with these differences in membranous *v*s cytoplasmic localization of P-cad in OSCC with different G, no statistical association was found between P-cad expression and any variables (age, sex, G, T, N and Stage grouping) considered at baseline.

**Figure 2:**
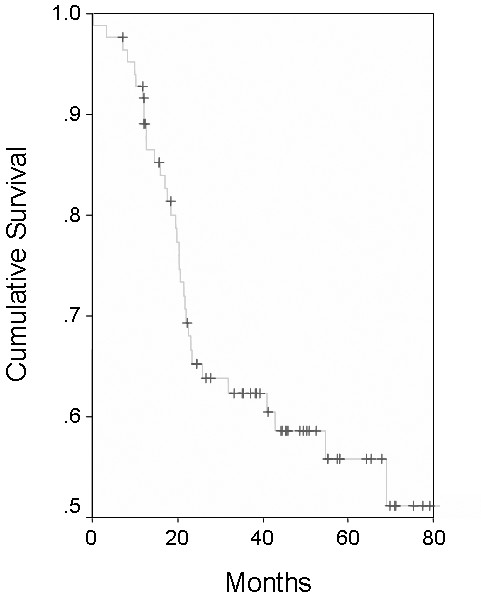
Overall disease-specific survival rate at 72-months (+ censored cases).

**Figure 3:**
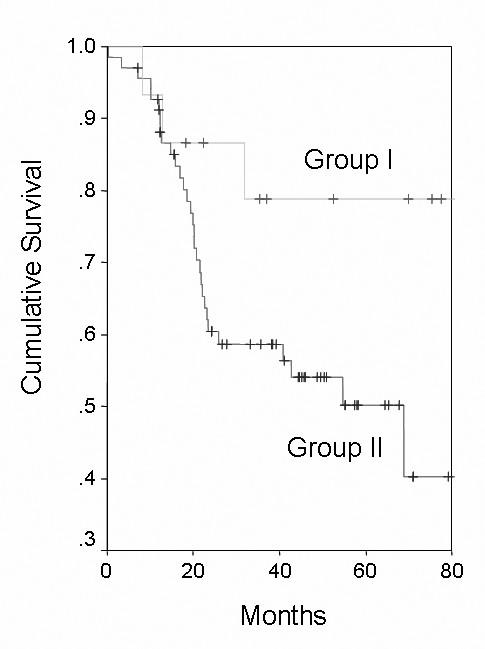
Disease-specific survival rate calculated according to P-cad categories. Log Rank 3.38, 1 df p-value = 0.0661 (+ censored cases)

The second part of the analysis planned the study of survival rates with respect to P-cad expression. Although the global disease-specific survival rate at 72 months was 51.0 %, irrespectively of the extent of the tumour or treatment (Fig. 2), the survival rates in the same cohort distributed according to P-cad expression (Group P-cad +ve vs. P-cad -ve) was 79.0% vs 40.0% respectively *(p-value = 0.04 Log Rank Test). *Survival curves stratified according to P-cad expression are illustrated in Fig 3. Still in terms of overall survival, within P-cad +ve group, OSCCs (n = 12) with a prevalent citoplasmic pattern of P-cad showed poorer survival rates than those (n = 25) with a prevalent membranous P-cad expression (P <0.0001). Besides the classic parameters related to survival rate and predictor of a poor outcome (e.g. G, T, N, stage and recurrence), a stepwise model introducing *P-cad *non-expression, without considering recurrence (parameter with the highest OR), showed that also *P-cad *non expression is significantly associated to survival, together with grading and stage. (Table 2, 3).

## Discussion

Both E- and P-cad play a pivotal role in the maintenance of the epithelial structure, even if they are expressed in distinct regions of the epithelium. E-cad is expressed on all epithelial layers, while P-cad is predominantly expressed in the basal layer of stratified squamous epithelia, the proliferative compartment [54-59]. E- and P-cad expression is altered in premalignant and malignant skin tumors, as demonstrated by reduced E-cad and aberrant P-cad expression in human squamous cell carcinomas [59], indicating the importance of coordinated cadherin expression for maintaining normal epidermal structure [60]. Malignant keratinocytes probably acquire different mechanisms for regulating the expression of these two cadherins [61]. P-cad seems to play a role in the maintenance of the epithelial phenotype and may be involved, together with E-cad, in the final stage of tumor progression in epidermal carcinogenesis, being a marker of hyperproliferative activity [47]. Studies on epidermis [26], gastric epithelium [49], and mammary epithelium [62] showed that P-cad controls cell proliferation in these tissues. P-cad expression seems to be related to tumour progression in gastric [50] and gingival carcinomas [35], while its expression is higher in poorly differentiated than in well-differentiated lung carcinomas [23]. In particular, well-differentiated oral carcinomas showed P-cad expression similar to normal oral mucosa or up-regulated, while P-cad expression homogeneously reduced in low-differentiated oral squamous cell carcinomas or its localization shifted to the cytoplasm, in accordance with other studies on oral mucosa [39] or gastrointestinal mucosa [48,63]. Williams et al. (1988) reported a loss of membranous immunostaining at the periphery of the islands of carcinoma with a cytoplasmic immunostaining or a complete loss[39]. In contrast, towards the centre of the islands the more differentiated cells showed mild or moderate membranous staining in well- or moderately-differentiated carcinomas, reflecting the pattern seen in dysplasia [39]. Recently, also in oral premalignant lesions induced in rats it has been found that E-cad and P-cad have similar location of expression as in OSCC and that just P-cad aberrant expression could be a strong marker of carcinogen progression[64].

On the basis of the current knowledge on P-cad and of a previous research conducted by one of the center involved[52], in the present research our main goal was to conduct an *in vivo *study on the clinical outcome (e.g overall survival rate) of OSCC with respect of quality and quantity of P-cad expression. Hence, in terms of prognostic significance, the lack of P-cad expression (44.8%) was found to have an independent association with poorer overall survival rate than P-cad expressing group; moreover, the abnormal (cytoplasmic) expression of P-cad is also associated to a poorer prognosis when compared to that normally membranous. In these latter cases, expression of P-cad shifted to membranous/cytoplasmic co-localisation, predominantly cytoplasmic in distribution, or alternatively was absent from a large numbers of cells. These two main results are consistent with a very recent Italian research [52] in which also other tissues (i.e. lymph node and bone metastases) were investigated.

As regards the lack of P-cad expression, it can be interpreted as a late event prior to invasion, as shown by loss of its expression in dysplasia adjacent to infiltrating carcinomas [39]. The loss of P-cad expression probably comes after P-cad cytoplasmic relocalization. Loss of P-cad expression in OSCC is associated with tumour invasion, while P-cad membranous staining in OSCC is probably due to the up-regulation seen in tumour cell lines and dysplasias. Therefore, in the initial phase of tumour growth the high expression of P-cad may be crucial in the formation of a tumour mass which is ready to progress and metastasize [50]. Whereas anomalous P-cad expression in the spinous layer of epithelium overlying tumour can be a biological marker for keratinocyte atypia and/or premalignant changes[65]. In fact, the continued expression of P-cad in the invasive cells can contribute to the maintenance of the epithelioid phenotype of the carcinoma cells [65]. An experimental study on squamous cell carcinoma cell lines showed aberrant expression in cancer cells, whereas E-cad expression was reduced [61]. SCC cells probably acquire the ability to express P-cad and this molecule plays a role in tumour progression [61]. Elevated [Ca^++^] determined increased cell-surface P-cad expression in SCC cell lines by up-regulation of de novo P-cad synthesis, while in normal keratinocytes calcium-induced cell-surface P-cad expression is a result of the translocation of pre-formed P-cad from the cytosol without up-regulation of P-cad synthesis [60]. These results suggest the existence of a unique mechanism for regulating the P-cad expression gene in tumour cells.

Bagutti et al. (1998) showed no correlation between P-cad expression and differentiation of tumour cells[40], while Sakaki et al. (1994) showed a complete loss of P-cad expression in poorly-differentiated gingival SCC[35]. Cytoplasmic relocalization or loss of P-cad expression may be responsible, together with other known/unknown upregulated oncogenes and downregulated tumour suppressor genes, for the later stages of tumour progression, such as invasive growth and metastasis [50].

On the basis of our present results, no association was found between P-cad expression and any of parameters considered (age, gender, G, T, N, Stage grouping), datum not consistent with the Italian research cited above [52], probably due to the different sample size.

A limit of the present study could be the fact that assessment of P-cad expression was done according to two scores; a larger sample size is warranted in the future in order to score OSCC in several groups according to P-cad expression with respect to prognosis.

The main present findings, based on overall survival rate, emphasise the role of P-cad expression as an early marker of OSCC prognosis, earlier than recurrence, as being the best marker, unfortunately, detected only after its onset.

## Competing interests

The author(s) declare that they have no competing interests.

## Authors' contributions

LLM conceived of the study, participated in its design- coordination and helped to draft the manuscript. GC participated in the design of the study, helped to analyse data and to draft the manuscript. AF participated in the design of the study and performed the statistical analysis. CS performed immunohistochemical study. RS revised the article critically. GL made substantial contributions to acquisition, analysis and interpretation of data. GP performed immunohistochemical study. ADL performed immunohistochemical study. FC has been involved in drafting the article. All authors read and approved the final manuscript.

## Pre-publication history

The pre-publication history for this paper can be accessed here:



## Figures and Tables

**Table 1:** OSCC grouped by P-cad expression and their features.

**Variables**	**No.**	**P-cad-ve (%)**	**P-cad+ve n. (%)**	**Mean**	**Standard deviation**	**P < 0.05**
Cases	67	30(44.8)	37 (55.2)			

Age						
</= 65 years	32	16	16	1.40	1.10	P = 0.363°
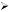 65 years	35	14	21	1.65	1.13	

Sex						
Male	45	20	25	1.55	1.07	P = 0.253°
Female	22	10	12	1.50	1.22	

Grading						
G1	21	6	15	1.85	1.10	P = 0.143*
G2	30	15	15	1.53	1.07	
G3	16	9	7	1.12	1.14	

Staging						
I	30	10	20	1,667	0,4795	P = 0,993*
II	15	6	9	1,600	0,5071	
III	11	7	4	1,364	0,5045	
IV	11	4	7	1,636	0,5045	

°Student-Newmann-Keuls' test.

*One-way Analysis of Variance (ANOVA) and Student-Newman-Keuls Multiple Comparisons Test

**Table 2:** Cox regression for survival rate with all variables known, apart from P-cad.

Variables	OR	95% CI		p-value
		Lower	Upper	

Grading	2.341	1.292	4.241	.005
T	.391	.162	.940	.036
N	.194	.062	.607	.005
Stage	6.553	2.327	18.456	.000
Recurrence	23.170	7.697	69.745	.000

**Table 3:** Cox regression analyses forced for the presence of P-cad and without considering recurrence.

Varziables	OR	95.0% CI		p-value
		Lower	Upper	

P-cad	4.068	.964	17.172	.056
Grading	1.942	1.164	3.240	.011
Stage	1.461	1.114	1.915	.006
